# Partial Sets of Teeth Sustained by Air Chambers Instead of Clasps

**Published:** 1851-04

**Authors:** A. Berry

**Affiliations:** Raymond, Miss.


### PARTIAL SETS OF TEETH SUSTAINED BY AIR CHAMBERS INSTEAD OF CLASPS.

BY A. BERRY, D.D.S., RAYMOND, MISS.

Cases in which it is desirable to sustain partial sets of teeth without clasps frequently occur. There may be no teeth healthy enough to warrant the attempt to retain others by them; or, if free from disease, their organization may be so defective as plainly to indicate that if clasps are thrown around them, caries will inevitably ensue in a few years at farthest. Is it not a fact, that in a majority of instances, the dentist of experience and sound judgment considers the teeth to which clasps are applied as sacrificed? It often happens that we are required to insert plate teeth for persons in early life. How greatly is our pleasure lessened in such cases by the reflection, that while we gratify our patient by furnishing gems highly prized by him, we cause the ultimate loss of more of his natural organs. This has been a matter of necessity until within a few years past, but the discoveries in dental science fortunately supply a remedy for this evil. By the aid of air chambers we may now support pieces with any required number of teeth attached, when the use of clasps is considered objectionable. Although they may not in all cases retain the pieces so firmly as clasps, they will generally, if properly made, answer very well and afford satisfaction to the patient, who will, of course, prefer to submit to some inconvenience, if necessary, to preserve other teeth from ruin.

The perusal of Dr. Dwinelle's article on air chambers, and the description of the valve he had with great ingenuity invented for them, in the American Journal of Dental Science, for January, 1850, was highly gratifying to me. At the time of its reception I was constructing a piece to support four incisors with Dr. Cleaveland's chamber, I resolved to apply Dr. Dwinelle's valve, and did so with the most satisfactory result. A few weeks afterwards I applied the valve again to a piece with four incisors attached, and as before was delighted with its success.

In conversation with several of my professional brethren subsequently, they stated, that in their opinion the valve was useless, while I thought otherwise. One of my patients after wearing her teeth several months lost the valve, and when informed of it, to test the question of its utility, I filled the opening for it with wood, and when several days had intervened, I was informed by her that the teeth were held *in situ* as firmly as before. This intelligence was very gratifying, as the construction of the valve and spring is an exceedingly nice and difficult work. A short time after this I made another piece with four incisors, to be sustained by Dr. Cleaveland's chamber without the valve, and when it was inserted and the air exhausted, it was held so firmly, that the patient found his strenuous efforts to remove it unavailing, and I was compelled to insert an instrument under the edge of the plate to pull it down. It was retained with as much force as it could have been with a valve. I would remark that these pieces were constructed before I was aware that Dr. Cleaveland had obtained a patent for his air chamber. Instead of leaving the space between the plates open according to Dr. C's mode, I packed it full with gold foil and soldered it.

I recently made a plate for two teeth with Mr. Gilbert's chamber, which was sustained firmly and promises to do well. I built upon the plaster cast to form the prominence on which to strike up the chamber, with plaster of Paris of the consistence of thin cream applied with a camel's hair pencil, prefering it to whiting from its drying sooner, and to beeswax, from the solution of the shellac drying on it and preventing the sand from adhering to it as it often does to the wax. In fitting the plate I hammered it closely down around the border of the chamber on one of the metal casts with a small blunt pointed instrument.

Dr. Cleaveland says in his specification, "The device that most nearly approximates to mine is that of Gilbert in which there is a depression in the center of a plate that like mine covers a portion of the palatine arch, but this when the air is withdrawn does not have an air chamber, the whole of the concavity being filled with the mucous membrane, and consequently there is less security in the attachment than in mine with the large annular air chamber in it.

The charge of a want of "security" in the attachment of Gilbert's chamber from its "being filled with the mucous membrane" is not verified in my experience with it, and I have never heard it urged by my friends, some of whom have applied it in a large number of cases. I do not advocate the general substitution of chambers instead of clasps, but that they should be resorted to when the condition or organization of the natural organs is such as to render it advisable. But it is always well to prepare the patient for the worst that may occur with it; or at least to be careful that he does not expect too much. No harm results from artificial teeth succeeding better than he supposes possible, while if his expectations are not realized he is never satisfied.

In only one instance have I known the mucous membrane to be drawn into the cavity so as to induce a slight inflammation and soreness—this however not to prevent the constant wearing of the teeth—and of but few days continuance. In all the cases in which I have used it the patients are satisfied, and the pieces with the exception of one, in which the form of the mouth was not the best, are retained in place with a good degree of firmness.

February 20th, 1851.

